# Beta-Blocker Therapy After Acute Myocardial Infarction

**DOI:** 10.3390/jcm15197583

**Published:** 2026-09-29

**Authors:** Chiara Cosentino, Luigi Cutore, Carmelo Raffo, Claudio Laudani, Carmelo Cusmano, Marco Spagnolo, Davide Capodanno, Antonio Greco

**Affiliations:** Division of Cardiology, Azienda Ospedaliero-Universitaria Policlinico “G. Rodolico—San Marco”, University of Catania, Via Santa Sofia, 78, 95123 Catania, Italycusmanocarmelo@gmail.com (C.C.);

**Keywords:** acute myocardial infarction, beta-blockers, cardiovascular pharmacotherapy, heart failure, secondary prevention

## Abstract

Historically, beta-blockers have long represented a cornerstone of secondary prevention after acute myocardial infarction (AMI). However, the transition to the modern era of early reperfusion and potent pharmacotherapy has questioned their universal efficacy, particularly in patients without post-AMI left ventricular dysfunction. This review critically analyzes current evidence on the role, timing, and optimal duration of beta-blocker therapy after AMI, with a focus on the impact of left ventricular ejection fraction (LVEF). Relevant literature was identified through searches of PubMed and Web of Science, with particular emphasis on randomized trials and meta-analyses investigating beta-blocker efficacy and safety post-AMI. The search strategy focused on the interaction between LVEF categories and clinical outcomes. Data from historical cohorts and modern trials were synthesized to evaluate the optimal timing and duration of therapy according to the LVEF phenotype. While the survival benefit of beta-blockers remains undisputed for patients with LVEF < 40%, contemporary evidence demonstrates a lack of prognostic advantage for routine beta-blockade in patients with preserved LVEF (i.e., ≥50%). Crucially, recent meta-analyses reveal that patients with LVEF (40–49%) may derive significant benefit from long-term therapy with beta-blockers. Regarding duration, evidence on deprescription remains conflicting. Although earlier data highlighted risks of hemodynamic rebound upon withdrawal, recent trials demonstrate that discontinuing beta-blocker therapy in the chronic phase of AMI may be a safe strategy. Long-term beta-blocker therapy after AMI is supported by robust evidence in patients with LVEF < 40%, whereas emerging but less definitive evidence suggests a potential benefit in those with LVEF 40–49%. Conversely, contemporary randomized trials do not support routine beta-blockade for patients with preserved LVEF (≥50%) in the absence of other clinical indications. As such, further studies are needed to refine patient selection and inform future guideline recommendations across the whole LVEF spectrum.

## 1. Introduction

Acute myocardial infarction (AMI) represents a leading cause of morbidity and mortality worldwide, placing a significant strain on healthcare systems: in the United States alone, an individual suffers an AMI approximately every 40 s, accounting for an estimated annual burden of over 800,000 new and recurrent events [1,2]. However, over past decades, the management of AMI has undergone a transformative shift from conservative strategies to the modern standard of early revascularization [3,4,5]. This evolution, combined with the introduction of contemporary drugs for secondary prevention, has significantly improved patient prognosis [6,7,8,9,10].

Historically, beta-blockers held a preeminent status as the cornerstone of secondary prevention following AMI [11,12,13,14]. Early randomized trials documented that long-term treatment with timolol and propranolol reduced mortality and the rate of reinfarction in AMI survivors [15,16]. Furthermore, a landmark meta-analysis from the late 90s established that the survival benefit was primarily driven by long-term administration of beta-blockers rather than their use in the acute phase alone, reporting a 23% reduction in mortality. Consequently, guidelines recommended lifelong beta-blocker therapy for all AMI survivors in the absence of absolute contraindications [17]. Nevertheless, as most of these trials have been conducted before the widespread adoption of primary percutaneous coronary intervention (PCI), extrapolating their results to contemporary practice comes with important limitations. 

Over the decades, left ventricular ejection fraction (LVEF) has been identified as a key determinant for stratifying the therapeutic benefit of beta-blocker therapy [18,19,20]. While the efficacy of beta-blockers in the high-risk subgroup of patients with LVEF < 40% was established, evidence supporting their routine use in patients with LVEF > 40 is less clear [21]. The paucity of robust evidence led to divergent recommendations, with American guidelines endorsing universal beta-blocker use regardless of LVEF and European guidelines supporting their adoption in patients with LVEF < 40%, thus highlighting the critical need for contemporary trials. In this context, several large-scale randomized trials have recently been conducted to re-evaluate the role of beta-blockers in the modern era [22,23].

The aim of this review is to critically analyze high-quality evidence from trials and meta-analyses to define the current role, timing, and optimal duration of beta-blocker therapy across the whole spectrum of LVEF following AMI.

### Literature Search Strategy

This narrative review was informed by a literature search of PubMed and Web of Science from database inception to 31 August 2026.. Search terms included combinations of “beta-blocker”, “beta-adrenergic blockade”, “acute myocardial infarction”, “myocardial infarction”, “acute coronary syndrome”, “left ventricular ejection fraction”, “heart failure”, “mortality”, “cardiovascular outcomes”, “early administration”, “intravenous beta-blocker”, “timing”, “duration”, “discontinuation”, “withdrawal”, and “deprescribing”. Emphasis was placed on randomized controlled trials, meta-analyses and contemporary international guidelines addressing beta-blocker therapy after AMI. Relevant observational studies and landmark historical trials were also considered to provide clinical and historical context. Reference lists of selected articles were additionally screened to identify potentially relevant publications. No restrictions by language were applied.

## 2. Mechanism of Action and Cardiac Effects of Beta-Blockers

### 2.1. Cardiac Β-Adrenergic Receptors: Signaling Pathways and Modulation

**Figure 1:**
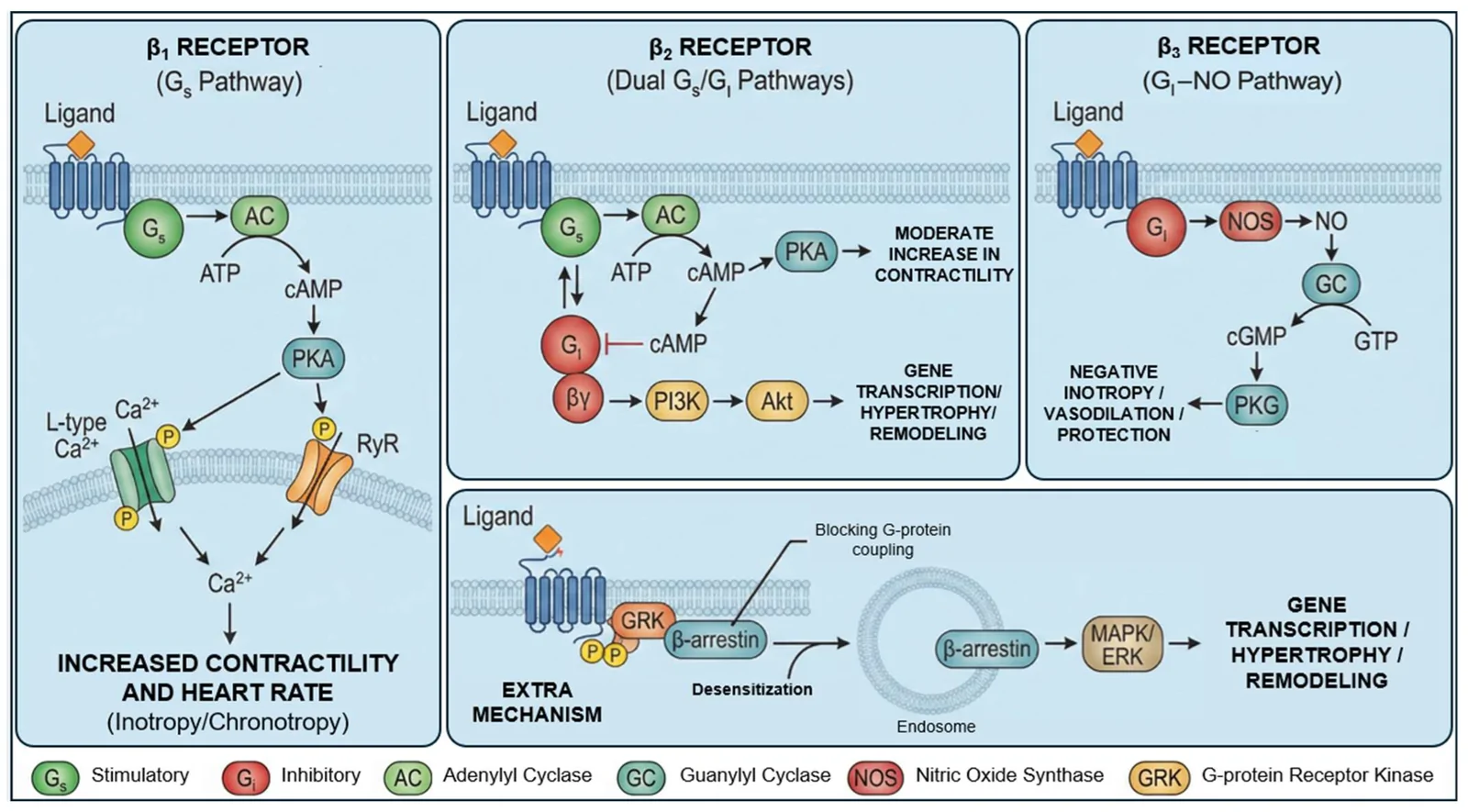
β-Adrenergic receptors in normal cardiac function and cardiac remodeling. Schematic representation of β_1_, β_2_, and β_3_ adrenergic receptor signaling in cardiac myocytes. β_1_ receptors couple to Gs, activating adenylyl cyclase, increasing cAMP, and stimulating PKA, which phosphorylates L-type Ca^2+^ channels, ryanodine receptors (RyR2), and phospholamban (PLB), thereby enhancing intracellular Ca^2+^ cycling, contractility, and heart rate. β_2_ receptors couple to both G_s_ and G_i_ in cardiac myocytes. G_s_ activation increases cAMP–PKA signaling, producing a spatially confined and moderate inotropic response. Following receptor phosphorylation, coupling to G_i_ attenuates adenylyl cyclase activity and recruits PI3K–Akt signaling, promoting pro-survival and adaptive remodeling pathways. β_3_ receptors couple to Gi–NOS signaling, increasing NO and cGMP–PKG activity, resulting in negative inotropy and cardioprotective effects. Receptor desensitization via GRK and β-arrestin, with downstream MAPK/ERK signaling, is also illustrated. Artificial intelligence tools were used to generate individual graphical elements; conceptualization and preparation of the figure did not involve artificial intelligence tools. Abbreviations: AC, adenylyl cyclase; Akt, protein kinase B; ATP, adenosine triphosphate; cAMP, cyclic adenosine monophosphate; cGMP, cyclic guanosine monophosphate; ERK, Extracellular signal-regulated kinase; GC, guanylyl cyclase; Gi, inhibitory G protein; GRK, G-protein-coupled receptor kinase; Gs, stimulatory G protein; GTP, guanosine triphosphate; MAPK, mitogen-activated protein kinase; NO, nitric oxide; NOS, nitric oxide synthase; PI3K, phosphoinositide 3 kinase; PKA, protein kinase A; PKG, protein kinase G; RyR, ryanodine receptor.

Adrenergic receptors include α1/α2 and β1/β2/β3 subtypes within the G protein-coupled receptors superfamily. In human cardiovascular physiology, β-adrenoceptors are the principal mediators of myocardial catecholamine effects, while α-adrenoceptors predominantly govern vascular tone and peripheral vasoconstriction (Figure 1) [24].

β1 receptors are quantitatively prevalent and couple primarily to G-alpha-s protein. Upon binding to β1 receptors, catecholamines trigger the activation of the adenylyl cyclase-cyclic adenosine monophosphate-protein kinase A axis, which phosphorylates L-type Ca^2+^ channels, phospholamban, troponin I, and ryanodine receptor 2 augmenting Ca^2+^ cycling and thereby inotropy, chronotropy, and lusitropy effects [25]. Against this signaling backdrop, β-blockers bind β1-adrenoceptors and competitively inhibit catecholamine activation, competitively limiting catecholamine-driven Gs activation and dampening downstream cyclic adenosine monophosphate-protein kinase A signaling [25]. Under conditions of sustained adrenergic stimulation—a pathological hallmark of heart failure (HF)—receptors undergo desensitization mediated by G protein-coupled receptor kinases phosphorylation and β-arrestin recruitment. This mechanism produces G protein uncoupling and receptor internalization, while also scaffolding mitogen-activated protein kinase/extracellular signal-regulated kinase signaling linked to hypertrophy and fibrosis. By reducing agonist-driven β1 activation, β-blockers decrease sustained receptor stimulation that promotes G protein-coupled receptor kinases-dependent phosphorylation and β-arrestin recruitment, thereby limiting receptor desensitization and internalization [24].

β2 receptors, frequently compartmentalized within specific membrane microdomains, can undergo a coupling switch toward G-inhibitory proteins to restrain cyclic adenosine monophosphate production. Clinically used nonselective β-blockers inhibit β2 receptors at the same orthosteric binding site, with the net effect of reducing adrenergic drive while potentially attenuating β2/G inhibitors “braking” that is typically recruited under conditions of stress [26].

Conversely, β3 receptors activate the counter-regulatory nitric oxide-cyclic guanosine monophosphate-protein kinase G axis and exert a protective negative inotropic influence. Conventional β-blockers have limited functional impact on β3 in vivo, such that this pathway is largely preserved during β1/β2 antagonism [24].

### 2.2. Clinical Pharmacology and Therapeutic Use of Β-Blockers

β-adrenergic receptor antagonists, commonly known as beta-blockers, are a pharmacologically heterogeneous class of agents that competitively inhibit the binding of endogenous catecholamines to β-adrenergic receptors, thereby attenuating sympathetic nervous system activity [27,28,29]. According to contemporary cardiovascular guidelines, beta-blockers remain a cornerstone therapy for HF, tachyarrhythmias, hypertension, and angina pectoris [30,31,32,33].

Most beta-blockers are administered orally to allow long-term treatment, whereas intravenous formulations are primarily employed in acute arrhythmic or perioperative settings [34,35]. Treatment should generally be initiated at low doses followed by gradual up titration to minimize excessive bradycardia, hypotension, or worsening HF [36,37,38,39,40]. As previously outlined, the cardiovascular sympathetic network relies on distinct receptor subtypes with specific tissue distributions: β1-adrenergic receptors predominate in the myocardium and renal juxtaglomerular apparatus, governing positive inotropic and chronotropic responses and renin secretion. Conversely, β2-receptors are primarily located in bronchial and vascular smooth muscle, promoting bronchodilation and vasodilation, while α1-receptors mediate peripheral vasoconstriction and hypertrophic cardiac remodeling [41,42]. From a physiological perspective, β1-receptor stimulation increases heart rate, myocardial contractility, atrioventricular conduction, and renin release, whereas β2-receptor stimulation promotes bronchodilatation, peripheral vasodilatation and also contributes to cardiac chronotropic and inotropic responses. Conversely, β1-receptor blockade reduces heart rate, myocardial contractility, atrioventricular conduction, and renin release, whereas β2-receptor blockade may attenuate bronchodilatation and peripheral vasodilatation, thereby increasing the potential for bronchoconstriction and peripheral vasoconstriction [43].

Significant pharmacodynamic differences exist among different beta-blockers, leading to their classification into three distinct generations based on receptor selectivity and ancillary properties (Table 1):

- First-generation agents (e.g., propranolol, nadolol, timolol, sotalol) are non-selective antagonists that block both β1 and β2 receptors, which may predispose patients to peripheral vasoconstriction and bronchospasm.
- Second-generation agents (e.g., bisoprolol, metoprolol, atenolol, esmolol) exhibit a preferential, dose-dependent affinity for β1-receptors. Due to their cardioselectivity, they carry a lower risk of triggering peripheral or bronchial constriction and are generally preferred in patients with chronic obstructive pulmonary disease or peripheral vascular disease.
- Third-generation agents (e.g., carvedilol, labetalol, and nebivolol) have additional vasodilatory properties beyond β-adrenergic blockade. Carvedilol and labetalol concurrently antagonize α1-receptors, thereby reducing systemic vascular resistance, whereas nebivolol promotes endothelial nitric oxide bioavailability through β3-receptor-mediated activation of endothelial nitric oxide synthase [44].

**Table 1:** Receptor selectivity, ancillary pharmacological properties, and clinically relevant effects of representative beta-blockers.

Agent/Class	Receptor Profile	Main Ancillary Mechanism	Main Clinical Implication
First-generation agents (propranolol, nadolol, timolol)	Non-selective β1/β2 antagonism [43,45]	Non-selective β-adrenergic blockade	Greater risk of peripheral vasoconstriction and bronchospasm [43,45]
Second-generation agents (bisoprolol, metoprolol, atenolol, esmolol)	Predominantly β1-selective * [45]	No major ancillary mechanism	Lower risk of peripheral and bronchial constriction; generally preferred in patients with COPD or peripheral vascular disease [45]
Carvedilol, labetalol	β1/β2 blockade with additional α1 antagonism [45]	α1-receptor blockade	Vasodilation and reduction in systemic vascular resistance [45]
Nebivolol	Highly β1-selective * [46]	β3-related enhancement of endothelial nitric oxide bioavailability	Additional endothelial vasodilatory effect [46]
Pindolol, acebutolol	β-receptor antagonism with intrinsic sympathomimetic activity [45]	Partial agonistic activity	Less pronounced resting bradycardia and smaller reductions in cardiac output; less complete adrenergic inhibition [45]

* β1-receptor selectivity is relative and dose-dependent; with increasing doses, selectivity becomes less pronounced and clinically relevant β2-receptor blockade may occur. Abbreviations: COPD, chronic obstructive pulmonary disease.

Another relevant pharmacological feature is intrinsic sympathomimetic activity, defined as partial agonistic activity despite competitive β-receptor antagonism [47,48,49,50]. Agents exhibiting intrinsic sympathomimetic activity, such as pindolol and acebutolol, are associated with less pronounced resting bradycardia and smaller reductions in cardiac output; however, they are currently less frequently employed in HF and post-AMI settings because they provide less complete adrenergic inhibition. Metabolic adverse effects, including weight gain, impaired insulin sensitivity, and erectile dysfunction, have also been reported, particularly with older non-vasodilating agents [51]. Importantly, although beta-blockers exert the same primary receptor-level antagonism in non-infarcted and post-AMI myocardium, their functional consequences differ according to the underlying myocardial substrate. In the non-infarcted heart, beta-adrenergic blockade predominantly reduces heart rate, contractility and myocardial oxygen demand, whereas after AMI these effects occur in the context of heightened sympathetic activation, residual ischaemia, electrical instability, and potentially impaired ventricular function. Accordingly, beta-blockade may reduce ischaemic and arrhythmic stress, but excessive negative inotropy may be particularly detrimental during the early post-AMI phase.

### 2.3. Beneficial Effects of Beta-Blockers in Heart Failure

In HF, chronic and persistent sympathetic overactivation serves as a foundational compensatory mechanism that ultimately drives progressive myocardial decay [47]. This sustained adrenergic barrage, mediated by excessive norepinephrine release from sympathetic nerve terminals and circulating epinephrine, paradoxically provokes a profound state of receptor desensitization [47]. At the molecular level, this impaired β-adrenergic responsiveness is mediated by two distinct pathological processes: receptor uncoupling and profound density downregulation [52]. Receptor uncoupling, structurally predominant within the β2-adrenoceptor subtype, occurs via a two-step biochemical cascade: upon continuous agonist occupancy, active receptors are phosphorylated by β-adrenergic receptor kinase, promoting the high-affinity binding of the cytosolic inhibitory protein β-arrestin. This interaction physically impairs coupling to G-proteins, blunting downstream signaling efficiency by up to 70%. Concurrently, receptor downregulation characteristically targets the β1-subtype, depleting the surface receptor pool through reduced messenger ribonucleic acid synthesis and accelerated lysosomal degradation. 

Crucially, the therapeutic efficacy of countering this impaired adrenergic system is strictly dictated by the individual LVEF. In HF with reduced ejection fraction (HFrEF), chronic sympathetic drive disrupts the delicate balance of intracellular calcium handling, shifting signaling away from efficient excitation-contraction and coupling toward maladaptive remodeling. Specifically, persistent adrenergic stimulation leads to a pathological intracellular Ca^2+^ overload, which serves as the primary trigger for the chronic activation of Calcium/Calmodulin-dependent protein kinase II; once hyperactivated, it phosphorylates key calcium-handling proteins, causing abnormal Ca^2+^ leakage from the sarcoplasmic reticulum. This electrical instability directly amplifies arrhythmogenesis, while the subsequent accumulation of Ca^2+^ within the mitochondria disrupts the organelle membrane potential, ultimately driving mitochondrial apoptosis and progressive cardiomyocyte loss [24,53]. Within the HFrEF cohort, beta-blockers break this toxic neurohumoral loop via competitive receptor antagonism, principally at β1 and variably at β2 depending on their selectivity. By mitigating catecholamine toxicity, they lower the phosphorylation burden on Ca^2+^-handling proteins, stabilize ryanodine receptor 2-dependent Ca^2+^ release, and mitigate triggered ventricular arrhythmias [54]. Over time, this proximal inhibition permits partial restoration of receptor responsiveness and neurohumoral rebalancing, translating into reverse remodeling and durable reductions in hospitalization and sudden death [53]. It must be noted that during initiation, acute β1-blockade exerts negative inotropic and chronotropic effects, while β2-antagonism induces peripheral vasoconstriction. This temporary interference with catecholamine support can transiently depress ventricular performance and exacerbate sodium retention, requiring low initial doses and gradual up-titration. Conversely, long-term therapy over three to six months counteracts chronic catecholamine cardiotoxicity, allowing myocardial recovery to overwhelm the initial cardiodepressant effects [54]. Thus, the clinical relevance of beta-adrenergic blockade is strongly time-dependent: during the acute phase of AMI, attenuation of sympathetic activation must be balanced against the risk of excessive negative inotropy, whereas after hemodynamic stabilization its rationale increasingly relates to modulation of chronic adrenergic activation and ventricular remodeling.

### 2.4. Adverse Effects and Contraindications

Bradycardia, hypotension, and conduction disturbances represent the most common cardiovascular adverse drug reactions in patients on beta blockers, particularly in patients with underlying cardiac risk factors [55,56]. Non-cardiovascular effects are also widely reported with beta-blockers, including fatigue, dizziness, gastrointestinal symptoms (e.g., nausea, constipation), and sexual dysfunction [57]. Absolute contraindications for the initiation of beta-blocker therapy include second- or third-degree atrioventricular block (in patients without a permanent pacemaker), critical limb ischemia, and bronchial asthma (specifically, when non-cardio-selective agents are considered), as well as known hypersensitivity to the drug [57]. Furthermore, clinical caution is mandated in patients presenting with severe HF, symptomatic bradycardia, or persistent signs of congestion as the negative inotropic properties of these agents can worsen HF, as well as the negative chronotropic effects can precipitate high-degree atrioventricular block and severe bradycardia [57]. Close monitoring for hypotension and potential drug–drug interactions—particularly with verapamil, diltiazem, digoxin, amiodarone, and ivabradine—is essential to ensure patient safety during therapy titration [54].

## 3. Guideline Recommendations

**Figure 2:**
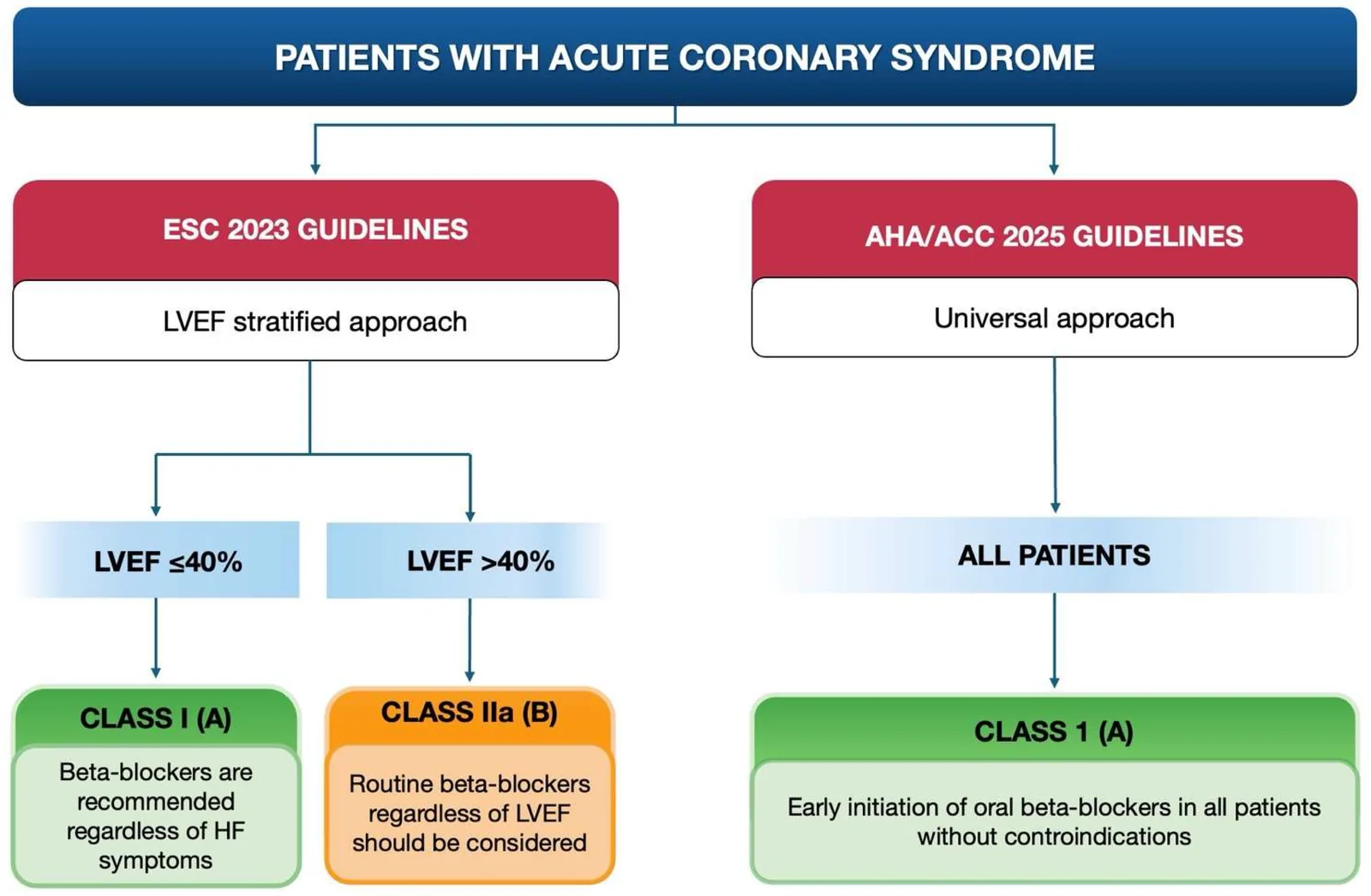
Comparison of guidelines recommendations for beta-blocker therapy after acute coronary syndrome. The diagram highlights the divergence between American and European guidelines in recommendations on beta-blocker therapy. In particular, European guidelines [3] endorsed an approach based on left ventricular ejection fraction, strongly mandating long-term beta-blocker therapy only for high-risk patients (LVEF ≤ 40%, Class I), with a weaker recommendation for those with an LVEF > 40% (Class IIa). Conversely, American guidelines [59] support a universal approach, mandating early oral initiation of beta-blocker therapy within the first 24 h of admission for all stable patients (Class 1), regardless of their baseline ejection fraction phenotype. A and B in parentheses indicate the level of evidence (LOE): A, Level of Evidence A; B, Level of Evidence B. Abbreviation: ACC, American College of Cardiology; AHA, American Heart Association; ESC, European Society of Cardiology; HF, heart failure; LVEF, left ventricular ejection fraction.

European and American guidelines present divergent recommendations for β-blocker therapy following acute coronary syndromes (ACS), reflecting the gap between established evidence from the pre-reperfusion era and the lack of robust randomized data in the modern era of early revascularization (Figure 2) [58].

According to 2023 European Society of Cardiology (ESC) guidelines [3], β-blockers are indicated with a class of recommendation (COR) I and a level of evidence (LOE) A in patients with LVEF ≤ 40%, even in the absence of HF symptoms. In this high-risk population, long-term β-blocker therapy is a cornerstone of secondary prevention, given its well established association with reductions in all-cause mortality, recurrent myocardial infarction, and ventricular arrhythmias [11,60]. Conversely, recommendations for routine beta blockade is less strong (COR IIa, LOE B) in patients with LVEF > 40%, reflecting the uncertain benefit in this population in the reperfusion era. Beyond this stratification, substantial uncertainties persist regarding the practical implementation of therapy. Specifically, ESC guidelines provide no clear consensus on the timing of beta-blockade initiation, and the optimal duration of treatment remains an open question. Although observational studies imply that the therapeutic benefit in uncomplicated ACS may be restricted to the first year, these findings are limited by potential bias due to the non-randomized designs of the data [61].

Conversely, the 2025 American guidelines [59], strongly recommend (COR 1, LOE A) the early initiation of oral beta-blockers within the first 24 h of ACS for all patients without contraindications, regardless of LVEF. Regarding the optimal duration of beta-blocker therapy, current guidelines on the management of chronic coronary syndromes emphasize that, although the strongest evidence for long-term beta-blockade remains firmly restricted to patients with LVEF ≤ 40% (COR 1, LOE A), its utility for reduction of major adverse cardiovascular events (MACEs) is less clear in patients with preserved LVEF. In particular, the clinical indication for long-term (>1 year) therapy should be reassessed after the first year in stable patients who were initially prescribed a beta-blocker after an AMI without a history of or systolic dysfunction (LVEF ≥ 50%), angina, arrhythmias or uncontrolled hypertension (COR 2b, LOE B-NR) [59]. More recently, the 2026 ESC guidelines for the management of HF [62], refined the categorization of HF according to LVEF with HFrEF defined as a LVEF < 50%. Of relevance to the post-AMI setting, beta-blockers are now recommended in patients with stage B (pre-HF) and LVEF < 50% to reduce the risk of HF hospitalization or death (COR I, LOE B1). In stable patients with symptomatic HFrEF, beta-blockers are also included as foundational medical therapy and are recommended to reduce HF hospitalization and death (COR I, LOE A). These recommendations therefore extend the therapeutic framework of beta-blockers across the LVEF 40–49% range in case of stage B or symptomatic HF criteria [62].

The impact of contemporary randomized trials on current recommendation is closely related to the timing of guideline publication. The 2023 ESC guidelines on ACS preceded REDUCE-AMI, ABYSS, REBOOT and BETAMI-DANBLOCK and therefore could not incorporate their findings, although they already acknowledged the uncertainty surrounding long-term beta-blocker therapy after uncomplicated ACS patients with LVEF > 40%. Conversely the 2025 ACS guidelines from the American College of Cardiology/American Heart Association (ACC/AHA) incorporated the evidence available from REDUCE-AMI, acknowledging the absence of demonstrated long-term benefit in revascularized patients with preserved LVEF, while maintaining the recommendation for early oral beta-blocker initiation in clinically stable patients. More recent trials, including REBOOT and BETAMI-DANBLOCK, have not yet been incorporated into guidelines and may therefore inform future recommendations.

Overall, contemporary guidelines integrate these considerations primarily by using LVEF and clinical stability to define long-term indications, while pathophysiological and hemodynamic considerations mainly inform the timing and safety of treatment initiation. However, current recommendations only partially incorporate the most recent randomized evidence, highlighting the need for further guideline updates as contemporary trials continue to accumulate.

## 4. Clinical Evidence for Beta-Blocker Therapy After Acute Myocardial Infarction

### 4.1. Early Evidence on Beta-Blocker Use After Acute Myocardial Infarction

The clinical use of beta-blockers after AMI was introduced during the pre-reperfusion era, following the results of several landmark trials [63]. Early randomized clinical trials (RCTs), such as the Beta-Blocker Heart Attack (BHAT) trial and the Norwegian Multicenter Study Group trial, demonstrated that long-term oral administration of non-selective agents successfully conferred a significant relative risk reduction in all-cause mortality (26% reduction with propranolol compared to placebo; 7.2% vs. 9.8%; *p* < 0.005), sudden cardiac death (45% reduction with timolol compared to placebo; 7.7% vs. 13.9%; *p* < 0.01) and recurrent ischemic event (14.4% reinfarction rate with timolol vs. 20.1% for placebo; *p* = 0.0006) [15,16].

Complementing these findings, the Gothenburg Metoprolol Trial tested the concept of early administration with a protocol of intravenous metoprolol (15 mg) immediately upon admission at the hospital followed by oral maintenance (100 mg twice daily) for 90 days; the trial reported a significant survival benefit with a 36% relative reduction in mortality in the metoprolol group compared to placebo (5.7% vs. 8.9; *p* = 0.030) [64]. Moreover, the ISIS-1 (First International Study of Infarct Survival) trial randomized more than 16,000 patients to a control group or to receiving atenolol (5–10 mg iv immediately, followed by 100 mg/day orally for 7 days) at a mean of 5 h after the onset of suspected AMI; intravenous atenolol resulted in a significant 15% relative reduction in vascular mortality within the first week (3.9% in the atenolol group vs. 4.6% in the control group; *p* < 0.04). Notably, a specific temporal analysis of the data revealed that the entirety of this survival benefit occurred within the first 24 h of treatment (1.5% vs. 2.1%; *p* < 0.01) [65]. However, this study also documented a significant increase in the need for intravenous inotropic agents (i.e., dopamine or dobutamine) in the treatment arm (5.0% vs. 3.4%; *p* < 0.01) [65]. This reflected early hemodynamic instability induced by acute beta-blockade, which was predominantly observed in vulnerable subgroups, such as patients presenting with a systolic blood pressure below 120 mmHg combined with tachycardia or atrial fibrillation.

The delicate balance between anti-ischemic efficacy and acute myocardial depression was further characterized by the COMMIT (Clopidogrel and Metoprolol in Myocardial Infarction (COMMIT) trial, being the largest trial evaluating over 45,000 patients who received an early intravenous dose of metoprolol (up to 15 mg) followed by oral maintenance, or matching placebo. The study found overall neutral results for the composite of in-hospital all-cause death, reinfarction or cardiac arrest (odds ratio [OR] 0.96; 95% confidence interval [CI] 0.90 to 1.01; *p* = 0.1). In addition, metoprolol significantly reduced the odds of reinfarction by 18% and the odds of ventricular fibrillation by 17%, while increasing the risk of cardiogenic shock by 30% [66].

Taken together, these historical data reveal a fundamental therapeutic paradox: while early beta-blockade provides undeniable anti-ischemic and anti-arrhythmic protection, its immediate negative inotropic effect poses a significant hemodynamic hazard. The systematic introduction of ultra-early intravenous regimens, as evaluated in ISIS-1 and COMMIT trials, established that the acute withdrawal of adrenergic support can precipitate pump failure, particularly in clinically vulnerable individuals presenting with borderline hypotension, tachycardia or Killip class ≥ 2. From a critical and a modern perspective, it is imperative to contextualize these findings within the era in which they were generated. The majority of these landmark trials were conducted prior to the routine implementation of contemporary myocardial revascularization and optimal secondary prevention strategies, including high-intensity statins and potent dual antiplatelets regimens [67]. In the current clinical landscapes, immediate mechanical reperfusion radically limits infarct size and preserves baseline LVEF, thereby diminishing the massive sympathetic overdrive that historically justified routine, unselected acute beta-blockade.

Consequently, the contemporary paradigm has shifted away from a reflexive, hyperacute intervention toward a highly stratified approach; in the modern reperfusion era, early routine intravenous administration is largely abandoned, and long-term maintenance therapy has been refined to target specific high-risk cohorts. The milestone transition into the reperfusion era was firmly consolidated by the Carvedilol Post-Infarct Survival Control in Left Ventricular Dysfunction (CAPRICORN) trial [68]. By focusing exclusively on clinically stable AMI patients with documented left ventricular systolic dysfunction (i.e., LVEF ≤ 40%), this trial randomized patients to carvedilol 6.25 mg or placebo, showing that third-generation beta-blockade with carvedilol significantly reduced the risk of the composite outcome of all-cause mortality, nonfatal myocardial infarction, or cardiac arrest compared to placebo (hazard ratio [HR] 0.58; 95% CI 0.38 to 0.91; *p* = 0.016) [68].

### 4.2. Contemporary Evidence in Patients with LVEF ≥ 40%

More randomized trials have recently emerged, specifically designed to re-evaluate efficacy of beta-blockers therapy in contemporary post-AMI care (Table 2). Among these, some studies specifically targeted patients with preserved LVEF (≥50%) whereas others adopted a broader inclusion criterion of LVEF > 40%. Notably, the latter inevitably included patients across the LVEF range of 40–49% and ≥50%. Therefore, the evidence derived from these specific studies reflects a mixed cohort rather than a specific subgroup with HFrEF and LVEF of 40–49%. The first contemporary evidence questioning the routine use of beta-blockers in this population came from the CAPITAL-RCT (Carvedilol Post-intervention Long-term Administration in Large-scale Trial) trial, a multicenter, open-label, randomized controlled trial which enrolled patients with a diagnosis of ST-segment elevation myocardial infarction (STEMI) undergoing successful primary PCI with a LVEF ≥ 40%; patients were randomly assigned within 7 days to receive carvedilol or no beta-blocker [69]. During a median follow-up of 3.9 years, the incidence of the primary endpoint (i.e., a composite of all-cause death, myocardial infarction, hospitalization for HF, and hospitalization for ACS) was similar between the two groups (HR 0.75; 95% CI 0.47 to 1.16; *p* = 0.20), as well as the occurrence of any coronary revascularization (HR 1.08; 95% CI 0.78 to 1.48; *p* = 0.65) [69]. This result led to a preliminary suggestion that the historical benefits of the beta blockade might not translate to a modern cohort of patients with LVEF ≥ 40%.

This hypothesis was tested in the REDUCE-AMI (Randomized Evaluation of Decreased Usage of Beta-Blockers after Acute Myocardial Infarction), an open label, randomized, multicenter trial enrolling 5020 patients 1–7 days after AMI and with preserved LVEF (i.e., ≥50%); patients were randomly assigned to the oral beta blockade group (at least metoprolol 100 mg or bisoprolol 5 mg) or no beta blockers; after a median follow-up of 3.5 years, long-term beta-blockade did not lead to a lower risk of death or new myocardial infarction compared to no treatment (HR 0.96; 95% CI 0.79 to 1.16; *p* = 0.64) [72]. Corroborating these findings, the REBOOT (Treatment With Beta-blockers After myOcardial Infarction withOut Reduced Ejection fracTion), an open-label, randomized trial evaluated the effect of beta-blocker therapy (type and dose determined by the treating physician) compared to no beta-blockers in patients with AMI and LVEF > 40%. REBOOT demonstrated that beta-blocker therapy failed to reduce the incidence of the primary composite outcome (i.e., death, reinfarction, or HF hospitalization) over a median follow-up of 3.7 years (HR 1.04; 95% CI 0.89 to 1.22; *p* = 0.63) [70].

Several methodological nuances and clinical factors may have contributed to the neutral results of these trials within the setting of patients with LVEF ≥ 40%. First, CAPITAL-RCT, REDUCE-AMI and REBOOT all utilized an open-label design which inherently introduces the risk of treatment crossover during long-term follow-up. In modern clinical practice, a significant proportion of patients initially randomized to the non-treatment arms are eventually prescribed beta-blockers for compelling concomitant indications, such as de novo arterial hypertension, palpitations, or supraventricular tachyarrhythmias. This inevitable contamination across cohorts tends to dilute the true therapeutic differences between the study arms, potentially masking a modest long-term benefit. Furthermore, these modern trials were conducted in populations characterized by an exceptionally low event rate, particularly in comparisons with earlier trials. Since contemporary secondary prevention strategies have substantially increased post-AMI survival, demonstrating an incremental mortality benefit with long-term beta-blockade would require either a larger sample size or a significantly extended follow-up period to achieve higher event rates and adequate statistical power.

A divergent result was obtained from the BETAMI-DANBLOCK (Betablocker Treatment After Acute Myocardial Infarction in Patients Without Reduced Left Ventricular Systolic Function and Danish Trial of Beta Blocker Treatment After Myocardial Infarction Without Reduced Ejection Fraction) randomized trial, enrolling 5574 patients who had an AMI and LVEF ≥ 40% [71]. Patients were randomized to receive beta-blocker therapy (the choice of type and dose were left to the discretion of the treating physician) or not within 1–3 days from the acute event and were followed up for a median time of 3.5 years [71]. In contrast to the previous studies, the investigators reported a statistically significant 15% reduction in the primary endpoint (i.e., a composite of death from any cause or major adverse cardiovascular events) in the beta-blocker arm (HR 0.85; 95% CI 0.75 to 0.98; *p* = 0.03) [71]. Importantly, the benefit was greater in patients with LVEF 40–49% than in those with LVEF ≥ 50%.

Of note, BETAMI and DANBLOCK were originally designed as two independent trials; however, due to slower than expected recruitment, they were formally combined into a single analysis using a harmonized primary endpoint. Despite this pragmatic adaptation, certain limitations should be highlighted: the cohort was characterized by a low proportion of female participants and metoprolol was predominantly used at a dose of 50 mg. These factors may restrict the generalizability of the study findings. A rigorous interpretation of these results would suggest that the predominant utilization of metoprolol at a lower maintenance dose of 50 mg/day significantly contrasts with the higher doses (e.g., metoprolol 100 mg) strictly mandated in the neutral REDUCE-AMI. Furthermore, the survival benefit of beta-blockers appeared selectively driven by the higher-risk subgroup, with a more evident treatment effect at lower LVEF values. Although differences in trial design, endpoint definitions, beta-blocker regimens, and LVEF ranges of enrolled patients may have contributed to these divergent findings, they cannot be assumed as the only determinant of the positive result of BETAMI-DANBLOCK.

Further complexity was added by the ABYSS (Assessment of Beta Blocker Interruption 1 Year after an Uncomplicated Myocardial Infarction on Safety and Symptomatic Cardiac Events Requiring Hospitalization) randomized noninferiority trial, which evaluated the safety of a strategy of interrupting beta-blocker therapy [73]. Notably, the ABYSS differs from previous studies by evaluating beta-blocker withdrawal rather than its initiation. Specifically, 3698 patients with previous myocardial infarction on long-term beta-blocker therapy (median time from index myocardial infarction to randomization 2.9 years) and LVEF ≥ 40% were randomized to either continue or discontinue the beta-blocker medication; at a median follow-up of 3.0 years, beta-blocker discontinuation was not noninferior to continued therapy, and was also associated with an increase in the composite of death, myocardial infarction, stroke, or hospitalization for cardiovascular reasons at 4 years (HR 1.16; 95% CI 1.01 to 1.33; *p* = 0.44 for noninferiority) [73]. More recently, SMART-DECISION (Smart Angioplasty Research Team: Decision on Medical Therapy in Patients with Coronary Artery Disease or Structural Heart Disease Undergoing Intervention) provided additional evidence on beta-blocker discontinuation using a different trial design. Reinforcing current American guidelines suggesting that the indication for long-term beta-blocker therapy in uncomplicated post-AMI patients with normal LVEF should be re-assessed over time, this study demonstrated that withdrawing beta-blockers after at least one year of treatment is noninferior to continuing therapy in patients with LVEF > 40% (HR 0.80; 95% CI, 0.57 to 1.13; *p* = 0.001 for noninferiority) [74]. While both studies reported low and comparable rates of hard clinical events between the groups, the inclusion of softer endpoints, such as hospitalizations, in the primary composite endpoint of ABYSS may have contributed to its negative results as, in the context of an open-label design, this specific endpoint remains more susceptible to influence from physician and patient awareness of drug withdrawal. Consequently, contemporary evidence highlights that the perceived safety of a discontinuation strategy largely depends on whether it is evaluated against hard or soft ischemic endpoints. However, ABYSS and SMART -DECISION differed not only in endpoint definition, but also in the enrolled populations and adopted noninferiority margins, thus limiting direct comparison between their findings. Importantly, both ABYSS and SMART-DECISION were designed as noninferiority trials, meaning that their findings should be interpreted within the constraints of a prespecified noninferiority margin [75]. Accordingly, failure to demonstrate noninferiority does not prove that treatment discontinuation is inferior but rather indicates that a clinically relevant excess risk could not be excluded.

### 4.3. Insights from Meta-Analyses

To provide definitive answers amidst these conflicting data, two separate individual patient-data meta-analyses were published. The first, pooling data from 17,801 patients with an LVEF of at least 50% enrolled in five randomized trials (i.e., CAPITAL-RCT, REBOOT, REDUCE-AMI, BETAMI, DANBLOCK), resulted in a similar incidence of a primary endpoint event (i.e., death from any cause, myocardial infarction, or HF) between patients receiving (8.3%) or not beta-blockers (HR 0.97; 95% CI 0.87 to 1.07; *p* = 0.54) [22]. Crucially, this lack of benefit was consistent across all prespecified subgroups—including age, sex, and type of infarction (STEMI vs. non-ST-segment elevation myocardial infarction)—and for all the individual components of the primary endpoint, confirming that routine beta-blockade offers no prognostic advantage in this population [22]. 

Conversely, the second meta-analysis focused on the specific group of 1885 patients with LVEF 40–49% from the REBOOT, BETAMI, DANBLOCK and CAPITAL-RCT trials [23]. In this setting, beta-blocker therapy was associated with a significant reduction in the composite of death, myocardial infarction, and HF (HR 0.75; CI 0.58 to 0.97; *p* = 0.031) [23]. Although individual components of the primary endpoint sometimes failed to reach statistical significance, mostly due to limited statistical power, the robust benefit observed in the composite outcome suggests a potential benefit of beta-blocker therapy in patients with LVEF 40–49%. However, these finding should be interpreted cautiously, as they derive from a relatively limited population and the 40–49% category reflects the predefined strata of the contributing trials rather than an established biological or therapeutic threshold. Accordingly, dedicated evidence is warranted before considering an update of current guideline recommendations.

The findings of these two meta-analyses provide a consistent framework of modern post-AMI beta-blockade across different LVEF ranges. Their value is significantly enhanced by the use of individual patient-level data, which, unlike traditional meta-analyses that merely aggregate effect sizes, enables the exploration of heterogeneity across the entire risk spectrum. However, it is important to note that these meta-analyses do not resolve all methodological and clinical debates. For instance, they do not address the optimal timing of beta-blocker initiation. Since trials varied significantly in their randomization windows, ranging from within 3 days to up to 7 days or more post-AMI, the specific prognostic impact of very early administration during the acute phase remains unclear and warrants further dedicated studies. Furthermore, concerns have been raised regarding the pragmatic, open-label design of these contemporary trials and the potential for lower treatment adherence compared to historical trials.

Regarding the increasingly debated issue of deprescription, a recent pooled individual patient-data analysis combined the two major randomized trials addressing this question, ABYSS and SMART-DECISION [76]. Using a one-stage mixed-effects Cox proportional hazard model, beta-blocker discontinuation met the prespecified criterion for noninferiority for the primary composite endpoint of all-cause death, myocardial infarction, stroke, or cardiovascular hospitalization (HR 1.09; 95% CI 0.97 to 1.24; *p* = 0.016 for noninferiority) [76]. However, this findings warrants cautions interpretation, as the upper limit of the 95% CI was very close to the prespecified noninferiority margin of 1.25 and the endpoint was largely influenced by cardiovascular hospitalizations, whose definitions were trial-defined. Conversely, noninferiority was more robustly demonstrated for the key secondary composite endpoint of all cause death, myocardial infarction, or HF hospitalization (HR 1.01; 95% CI 0.83–1.23; *p* = 0.0006 for noninferiority), which was less susceptible to bias related to the open-label design [76]. Overall, findings from this recent analysis provide additional support for considering late beta-blocker discontinuation in appropriately selected stable patients.

## 5. Optimal Timing and Duration of Beta Blockers Therapy

Another critical issue regarding beta receptor blockade is represented by the optimal timing and duration of beta blocker administration. Indeed, while early beta blocker initiation and lifelong therapy were encouraged based on studies from the pre-reperfusion era, subsequent evidence from large randomized trial challenged this dogma (Table 3) [77,78,79,80,81].

From a clinical perspective, three distinct therapeutic questions should be separately considered: acute initiation of beta-blocker therapy after AMI, its long-term continuation, and late withdrawal in clinically stable patients. Importantly, these strategies have been evaluated in different settings and should not be considered interchangeable.

With regard to the timing of intervention, there is currently no trial directly comparing early or delayed beta blockade, although some data can be inferred by trials of beta-blocker administration in AMI. Collectively, findings from initial trials highlighted that early, aggressive beta-blocker administration represented a complex clinical trade-off [65]. On one hand, the biological rationale is compelling as sympathetic overactivity acutely elevates myocardial oxygen consumption and exacerbates ischemic damage in the hyperacute phase of AMI. By mitigating this adrenergic storm, beta-blockers reduce oxygen demand and mediate direct cardioprotective effects. On the other hand, immediate implementation of beta-blockers is not always feasible due to the potential for unstable hemodynamics in the very acute phase of AMI. Although beta-blockers are generally initiated early after AMI, their administration should be guided by the patient’s hemodynamic status. Current ACC/AHA recommendations support initiation of oral beta-blocker therapy within the first 24 h in clinically stable patient without acute HF, low-output state, increased risk of cardiogenic shock, or other contraindications. In patient presenting with hemodynamic instability or other temporary contraindications, treatments should be deferred and reconsidered once clinical stability has been achieved.

Subsequent randomized trials evaluated whether the timing of intravenous administration—either prior to or immediately following mechanical reperfusion—could optimize myocardial salvage without compromising safety. The METOCARD-CNIC (Effect of Metoprolol in Cardioprotection During an Acute Myocardial Infarction) trial demonstrated that administering intravenous metoprolol in the ambulance or at the PCI center before revascularization significantly reduced infarct size by approximately 20% at magnetic resonance imaging compared to no beta blockade (adjusted difference −6.52; 95% CI −11.39 to −1.78; *p* = 0.012), achieving a higher long-term LVEF (adjusted difference 2.67%; 95% CI 0.09 to 5.21; *p* = 0.045) without a significant difference in the 24 h safety composite of death, shock, or arrhythmias (*p* = 0.21) [83]. Conversely, the larger EARLY-BAMI (Early-Beta blocker Administration before reperfusion primary PCI in patients with ST-elevation Myocardial Infarction) trial reported neutral effects on infarct size, LVEF and cardiogenic shock, showing only a borderline reduction in malignant arrhythmias (*p* = 0.05) [84]. In the post-reperfusion phase, short-acting beta-blockers infusions evaluated in the BEAT-AMI (Beta-Blocker Therapy in Acute Myocardial Infarction) trial yielded neutral results regarding myocardial salvage, but confirmed a highly favorable hemodynamic profile with no episodes of cardiogenic shock in the beta-blocker arm compared to 6% in the placebo group [85]. Consolidating these data, a patient-pooled meta-analysis confirmed that early intravenous administration of beta-blockers improved 6-month LVEF (52.8% vs. 50.0%; *p* < 0.01), without increasing a composite safety endpoint of shock, symptomatic bradycardia, or hypotension [79].

The striking divergence in cardiogenic shock rates between historical and contemporary trials is strictly driven by patient selection and the implementation of mechanical reperfusion [87,88,89]. Reflecting these favorable safety data on early initiation, in the absence of high-quality evidence on the timing of oral beta-blocker administration, American guidelines explicitly support a proactive stance, encouraging beta-blocker assumption within the first 24 h of admission for hemodynamically stable patients [58]. In contrast, European guidelines do not express a specific timing threshold, presumably acknowledging that larger definitive trials are still warranted to fully clarify the systematic efficacy and safety of early beta-blockage in current practice [3].

Regarding optimal duration of beta-blocker therapy, it should be noted that cardiovascular risk following AMI is not steady but evolves over time, therefore requiring continuous risk stratification and subsequent therapeutic adjustments [90,91]. In particular, recommendations can depend on the baseline LVEF and can be inferred from the duration of therapy in the aforementioned individual patient-data meta-analyses. Indeed, given the positive results up to 4 years in patients with LVEF 40–49%, it is safe to conclude that in this population beta-blockers may be safely administered up to 4 years, and, given the continuous divergence of the curves, longer continuation may still be beneficial [71]. Conversely, there is lack of evidence in patients with preserved LVEF (≥50%), where beta-blocker duration should be defined on a patient-to-patient basis [22,23].

Finally, the concept of deprescription—the systematic process of withdrawing medication or reducing dose where benefit is no longer evident to improve outcomes and reduce costs—has gained significant momentum in cardiovascular medicine [92,93,94,95,96]. Until recently, data on beta-blocker discontinuation relied exclusively on the ABYSS trial, which offered a crucial cautionary note, demonstrating that withdrawing beta-blockers in patients with LVEF ≥ 40% failed to meet noninferiority compared to continuation, a result largely driven by increased hospitalization for cardiovascular reasons caused by a rebound effect on heart rate or blood pressure [73]. SMART-DECISION established the noninferiority and safety of beta-blocker discontinuation after at least one year from AMI in patients with LVEF > 40%, when considering a harder composite endpoint of death, myocardial infarction, and HF hospitalization [74]. However, the two trials differed in enrolled population, endpoint definitions, and noninferiority margins, thereby limiting direct comparison of their findings. This discrepancy is further complicated by the distinct demographic and geographical profiles of the study cohorts, with ABYSS enrolling patients in France and SMART-DECISION in South Korea, potentially reflecting regional differences in standard-of-care background therapies, inherent cardiovascular risk, or genetic variations in drug metabolism [97,98,99,100]. Taken together, these findings suggest that long-term beta-blocker therapy may not be universally required over the long term in stable post-MI patients with preserved LVEF, with decisions on discontinuation remaining individualized according to clinical characteristics and potential alternative indications for beta-blocker use [97,101].

## 6. Expert Opinion and Future Directions

The routine use of beta-blockers after AMI has been largely informed by evidence derived from trials conducted in a clinical context markedly different from contemporary practice, characterized by extensive areas of nonperfused myocardial necrosis and a high incidence of adverse ventricular remodeling and malignant arrhythmias [15,16]. In the modern era, the widespread adoption of primary PCI, technical advances and the systematic use of secondary prevention pharmacological therapies, has substantially reduced residual ischemic risk [102,103,104,105,106,107,108,109]. Consequently, the broad prognostic benefit historically attributed to beta-blockade may be attenuated, and its clinical efficacy is now better understood as heterogeneous and closely dependent on patient phenotype, with LVEF emerging as a key determinant of the therapeutic effect [72]. In patients with LVEF ≤ 40% or with overt clinical signs of HF, a survival benefit associated with beta-blocker therapy remains well documented [68]. By contrast, contemporary randomized trials have not demonstrated significant reductions in mortality or recurrent AMI in patients with LVEF ≥ 40% [70,72]. BETAMI-DANBLOCK yielded discordant results compared with REDUCE-AMI and REBOOT, which can partly reflect differences in trial design, endpoint definitions, beta-blocker regimens, and the LVEF range of populations enrolled. Indeed, while REDUCE-AMI restricted enrolment to patients with preserved LVEF (≥50%) and, similarly to REBOOT, evaluated a narrow composite of hard clinical outcomes, BETAMI-DANBLOCK included patients with an LVEF ≥ 40% and adopted a broader composite primary endpoint [70,71,72]. However, these methodological differences cannot exclude the possibility of a genuine treatment benefit in selected patients, particularly those with LVEF 40–49%. Taken together, these data suggest that the treatment effect of beta-blockers may vary along the LVEF continuum, with robust evidence at lower LVEF values and emerging evidence of benefit in the 40–49% range.

Meta-analyses based on aggregate individual patient-data have shown different treatment effects across trial-defined LVEF categories, with no measurable reduction in major cardiovascular outcomes among patients with preserved LVEF. However, these categories largely reflect the inclusion criteria of the contributing trials and should not be interpreted as establishing a discrete biological or therapeutic threshold at an LVEF value of 50% [22,23].

From a clinical perspective, beta-blocker therapy should be initiated after hemodynamic stabilization and continued over the long term in patients with LVEF < 40%, particularly in the presence of HF [23,66]. Emerging evidence suggests a potential benefit also in patients with LVEF 40–49%, although treatment decisions in this group should remain tailored over the individual patient. Finally, current randomized evidence is not sufficient to justify routine initiation of long-term beta-blocker therapy in unselected patients with preserved LVEF and no concomitant indications [22]. Conversely, in patients with LVEF ≥ 40% who are already receiving beta-blockers, treatment discontinuation should be individualized and cautiously approached [73,74].

While waiting for extended follow-up from randomized trials, parallel research continues to refine acute-phase protocols. For example, an ongoing mechanistic trial (NCT06967194) is evaluating whether a 72 h regimen of low-dose metoprolol can prevent progression to Society for Cardiovascular Angiography and Interventions (SCAI) stage C cardiogenic shock in STEMI patients presenting at stage B. In addition, further exploring the benefits of early acute-phase administration, a prospective observational cohort study is investigating the cardioprotective role of highly cardioselective agents (NCT07293832); this study is evaluating whether early intravenous administration of landiolol reduces the final extent of the necrotic area compared to standard oral beta-blocker therapy in STEMI patients undergoing primary PCI. Moving beyond acute intravenous administration, a clinical trial (NCT06562582) is currently evaluating the effects of immediate versus delayed initiation of beta-blockers, angiotensin converting enzyme inhibitors, and sodium-glucose co-transporter 2 inhibitors following STEMI in terms of adverse LV remodeling. Collectively, these ongoing studies are expected to clarify several unresolved aspects of acute beta-blocker therapy. NCT06967194 will provide information on whether early low-dose beta-blockade may prevent hemodynamic deterioration in STEMI patients at increased risk of cardiogenic shock, whereas NCT07293832 will explore whether very early intravenous administration of a highly cardioselective beta-blocker may reduce infarct size in patients undergoing primary PCI. NCT06562582 will further investigate whether immediate versus delayed initiation of beta-blockers influences early LV remodeling after STEMI. Importantly, these studies are primarily focused on acute-phase treatment and are not designed to resolve the separate questions of optimal long-term treatment durations or late beta-blocker withdrawal, which therefore remain important evidence gaps.

## 7. Conclusions

For decades, clinical guidelines have endorsed beta-blocker therapy as a class I recommendation for all AMI survivors. However, the transition to the modern reperfusion era and the advances in cardiovascular pharmacotherapy have transformed the landscape of AMI, requiring a re-evaluation of this historical dogma. A critical reading of available evidence leads to a more individualized therapeutic paradigm, which is based on LVEF. In particular, available data provide robust support for beta blocker therapy in AMI patients with LVEF < 40%, while emerging but less definitive evidence suggests a potential benefit also in the 40–49% range; conversely, contemporary evidence does not support the routine use of beta blocker therapy in patients with LVEF ≥ 50% and no concomitant clinical indications.

## Figures and Tables

**Table 2:** Key randomized clinical trials evaluating beta-blocker therapy in the reperfusion era.

Trial, Year	Population	Sample Size	Intervention	Control	Primary Endpoint	Main Findings
CAPRICORN, 2001 [68]	AMI and a LVEF ≤ 40%	1959	Carvedilol 6.25 mg	No beta-blocker therapy	All-cause mortality or hospital admission for cardiovascular reasons	Carvedilol reduced the rates of all-cause and CV death, and recurrent AMI
REBOOT, 2025 [70]	AMI and LVEF ≥ 40%	8438	Beta-blocker therapy	No beta-blocker therapy	Composite of death from any cause, recurrent AMI, or hospitalization for HF	Beta-blocker had no effect on all-cause death, recurrent AMI, or HF hospitalization
BETAMI–DANBLOCK, 2025 [71]	AMI and LVEF of at least 40%	5574	Long-term beta-blocker therapy	No beta-blocker therapy	Composite of death from any cause or MACE (AMI, unplanned coronary revascularization, ischemic stroke, HF, or malignant ventricular arrhythmias)	Beta-blocker therapy led to a lower risk of death or MACE compared to no beta-blocker therapy
REDUCE-AMI, 2024 [72]	AMI and LVEF of at least 50%	5020	Long-term beta-blocker therapy (metoprolol or bisoprolol)	No beta-blocker therapy	Composite of death from any cause or recurrent AMI	Long-term beta-blocker treatment did not reduce the risk of the composite primary endpoint
CAPITAL- RCT, 2018 [69]	STEMI patients undergoing successful PCI within 24 and with LVEF ≥ 40%	801	Carvedilol within 7 days after primary PCI	No beta-blocker therapy	Composite of all-cause death, AMI, HF hospitalization, or hospitalization for ACS	Long-term carvedilol did not reduce the risk of the primary endpoint
ABYSS, 2024 [73]	Patients with previous AMI, LVEF > 40% and no CV events in the previous 6 months while on beta-blocker therapy	3698	Beta-blocker discontinuation	Beta-blocker continuation	Death, nonfatal AMI, nonfatal stroke, or hospitalization for cardiovascular reasons	Beta-blocker discontinuation was not noninferior to beta-blocker continuation
SMART -DECISION, 2026 [74]	Stable patients at least 1 year after AMI, with LVEF ≥ 40% and without HF	2540	Beta-blocker discontinuation	Beta-blocker continuation	Composite of death from any cause, recurrent AMI, or HF hospitalization	Discontinuation of beta-blocker therapy was noninferior to beta-blocker continuation

Abbreviations: ACS, acute coronary syndrome; AMI, acute myocardial infarction; CV, cardiovascular; HF, heart failure; LVEF, left ventricular ejection fraction; MACE, major adverse cardiovascular event; PCI, percutaneous coronary intervention; STEMI, ST-segment elevation myocardial infarction.

**Table 3:** Trials evaluating timing of administration and duration of beta-blocker therapy in patients with acute myocardial infarction.

Trial, Year	Population	Sample Size	Intervention	Control	Primary Endpoint	Main Findings
ISIS-1, 1986 [65]	AMI within 5 h from admission to the CCU	16,027	Atenolol (immediate IV bolus of 5–10 mg followed by 100 mg/day orally)	No beta-blocker	All-cause death	Atenolol reduced all-cause and vascular mortality at 1 year, while increasing the use of inotrope drugs
COMMIT, 2005 [66]	Patients with suspected AMI within 24 h from onset	45,852	Metoprolol (IV bolus up to 15 mg followed by 200 mg/day orally until discharge or up to 4 weeks)	Placebo	(i) Composite of all-cause death, recurrent AMI, or cardiac arrest; (ii) all-cause death	Metoprolol did not reduce the risk of the co-primary endpoints
Hanada et al., 2011 [82]	STEMI patients within 12 h from symptom onset	96	Periprocedural IV landiolol (continuous infusion 3 mcg/kg/min for 24 h)	No beta-blocker	Incidence of cardiovascular events at 6 months	No significant difference in cardiovascular events
METOCARD-CNIC, 2013 [83]	STEMI patients within 4.5 h from symptom onset	220	Periprocedural IV metoprolol (up to 3 boluses of 5 mg each)	No beta-blocker	Infarct size assessed by CMR at 7 days	Metoprolol significantly reduced infarct size
EARLY-BAMI, 2016 [84]	STEMI patients within 12 h from symptom onset	683	Periprocedural IV metoprolol (twice bolus of 5 mg)	Placebo	Infarct size assessed by CMR	No significant differences in infarct size
BEAT-AMI, 2016 [85]	STEMI patients within 6 h from symptom onset	100	IV esmolol (weight-adjusted dose to target a 60 beat per minute heart rate) after admission to the CCU	Placebo	Maximum change in troponin T from baseline to 48 h	Esmolol significantly reduced troponin T levels at 48 h
PROTECTION, 2021 [86]	STEMI patients within 24 h from symptom onset	47	Periprocedural IV landiolol (continuous infusion 3 mcg/kg/min up to a total dose of 50 mg)	No beta-blocker	Myocardial salvage index at 7 days	Landiolol increased myocardial salvage index

Abbreviations: AMI, acute myocardial infarction; CCU, cardiac care unit; CMR, cardiac magnetic resonance; IV, intravenous; LVEF, left ventricular ejection fraction; STEMI, ST-segment elevation myocardial infarction.

## Data Availability

No new data were created or analyzed in this manuscript. Data sharing is not applicable to this article.
